# Epidemiology and clinical aspect of pediatric mushroom poisonings: a 15-year retrospective analysis

**DOI:** 10.3389/fped.2025.1621891

**Published:** 2025-09-18

**Authors:** ShaoCong Zheng, Xuejiao Miao, Shan He

**Affiliations:** ^1^ College of Medicine, Kunming University of Science and Technology, Kunming, China; ^2^ Department of Pediatrics, The First People’s Hospital of Yunnan Province, Kunming, China

**Keywords:** pediatric, mushroom poisoning, HOPE6 scoring system, epidemiology, clinical manifestations

## Abstract

**Background:**

Mushroom poisoning is a significant public health concern, particularly in pediatric populations, where developmental differences in toxin metabolism and organ vulnerability pose unique clinical challenges. Despite its geographic and seasonal patterns, pediatric mushroom poisoning remains underrepresented in the literature, necessitating further investigation into its epidemiological and clinical characteristics.

**Methods:**

This 15-year retrospective cohort study analyzed 73 pediatric cases (aged ≤14 years) of mushroom poisoning at a tertiary hospital in Southwest China. Epidemiological, clinical, and laboratory data were collected, and statistical analyses were performed using SPSS 26.0.

**Results:**

Epidemiological data revealed seasonal clustering in summer and autumn, predominantly affecting older children (≥7 years). *Boletus spp.* accounted for most cases (71.2%), followed by *Amanita* (8.2%). Clinical presentations included gastrointestinal (72.6%) and neurotoxic symptoms (69.9%), with hallucinations more prevalent in non-liver injury cases (56.9% vs. 0%, *p* = 0.02). Severe hepatic injury, marked by elevated liver enzymes (ALT, AST, LDH) and coagulation dysfunction (APTT, PT), correlated with higher HOPE6 scores (≥3) and 100% mortality (4 deaths). The HOPE6 scoring system demonstrated prognostic utility, with a pediatric-specific threshold (≥3) predicting adverse outcomes, contrasting with adult thresholds (≥2).

**Conclusions:**

This study highlights the critical role of hepatic injury in mortality and underscores age-dependent variations in clinical thresholds for risk stratification in pediatric mushroom poisoning. Early intensive care is advocated to improve outcomes. Future research should focus on multicenter prospective cohorts to further validate these findings and assess therapeutic interventions.

## Introduction

Mushroom poisoning is a significant public health concern worldwide, particularly in regions where wild mushroom foraging is culturally entrenched (1, 2). Accidental foraging and ingestion of these mushrooms can lead to acute poisoning, characterized by geographic and seasonal incidence patterns, often manifesting as family clusters or community outbreaks, posing significant public health risks (3, 4). Certain species exhibit high case fatality rates; for instance, hepatotoxic *Amanita spp.* are associated with a mortality rate exceeding 80%. The clinical presentation is highly heterogeneous (5, 6). Most patients initially experience gastrointestinal symptoms such as nausea, vomiting, abdominal pain, and diarrhea (7, 8). Subsequent manifestations depend on the specific toxins ingested, which may induce diverse target organ injuries (including hepatic, renal, or neurological failure) or death (2, 5, 9). Consequently, the early identification of life-threatening mushroom poisoning and the implementation of standardized treatment protocols remain critical challenges for healthcare providers. While global epidemiological patterns have been well-documented in adults, pediatric populations are underrepresented in the literature. Mushroom toxicity in children presents unique clinical challenges due to developmental differences in toxin metabolism, symptom progression, and organ vulnerability.

The HOPE6 scoring system is a critical tool for the initial assessment of patients with mushroom poisoning (10). It helps clinicians quickly identify potentially fatal cases by evaluating 4 key aspects (History (H), Organ Damage (O), Picture Identification (P), Eruption of Symptom >6 h (E6), specific criteria in Supplementary). Each of these 4 aspects is scored as 1 point. If the total HOPE6 score is ≥2 points, the patient is considered to have potentially fatal mushroom poisoning and requires immediate intensive care and bundled treatment. This scoring system enables early recognition and appropriate management of severe mushroom poisoning cases, improving patient outcomes (10).

This study aimed to characterize the epidemiological patterns of pediatric mushroom poisoning in Southwest China, and validate the prognostic utility of the HOPE6 score in children.

## Method

### Study design and setting

Approval from the institutional ethics committee was obtained. The need for informed consent was waived due to the retrospective nature of the study. This study conducted a retrospective analysis of children with mushroom poisoning treated at the First People's Hospital of Yunnan Province over a 15-year period. This tertiary referral center serving Southwest China, a region endemic to wild mushroom foraging due to its subtropical climate and forested terrain. The hospital is equipped with a specialized unit managing most of severe mushroom poisoning cases in the region. We included all records from January 1, 2010 to December 31, 2024. Inclusion criteria: 1. Documented history of mushroom ingestion with associated poisoning symptoms; 2. Pediatric patients aged ≤14 years. Exclusion criteria: Patients with pre-existing medical history of hepatorenal dysfunction, coagulation disorders, neurological disorders, cardiac diseases, or other metabolic disorders. We also excluded patients with incomplete medical records.

### Study population and data collection

The following parameters were extracted: date, age, sex, BMI, Hepatic Outcome Prediction Evaluation-6 score (HOPE6 score), mushroom species, syndrome, hospitalization length, relevant laboratory parameters (include Alanine Aminotransferase (ALT), Aspartate Aminotransferase (AST), Lactate Dehydrogenase (LDH), Total Bilirubin (TBIL), Direct Bilirubin (DBIL), Unconjugated Bilirubin (UBIL), Albumin (ALB), Creatine Kinase-MB Isoenzyme (CK-MB), Creatinine (Cr), Blood Urea Nitrogen (BUN), Uric Acid (UA), Activated Partial Thromboplastin Time (APTT), Prothrombin Time (PT), fecal occult blood (FOB) et.) and outcome.

### Statistical analysis

Statistical analyses were performed using SPSS 26.0. Quantitative data conforming to a normal distribution were expressed as mean and standard deviation (SD) and analyzed with Student's *t*-test for intergroup comparisons. For non-normally distributed data, the Mann–Whitney *U*-test (nonparametric rank-sum test) was applied. Qualitative data were presented as percentages (%), with intergroup differences assessed using the Chi-square test. Fisher's exact test was substituted when any cell in the 2 × 2 contingency table contained fewer than 5 observations. Differences were considered statistically significant at *p* < 0.05.

## Results

### Patient enrollment and demographics

**Figure 1:**
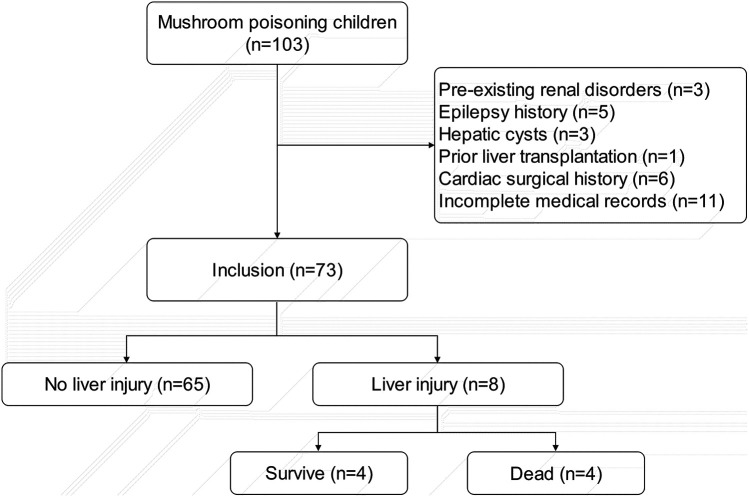
Patient enrollment flowchart.

Among 102 pediatric patients with documented mushroom ingestion and poisoning symptoms, 73 were ultimately enrolled in the study. Exclusions comprised: 3 patients with pre-existing renal disorders; 5 with epilepsy history; 3 with hepatic cysts; 1 with prior liver transplantation; 6 with cardiac surgical history; and 11 excluded due to incomplete medical records (Figure 1).

### Seasonal and age distribution

**Figure 2:**
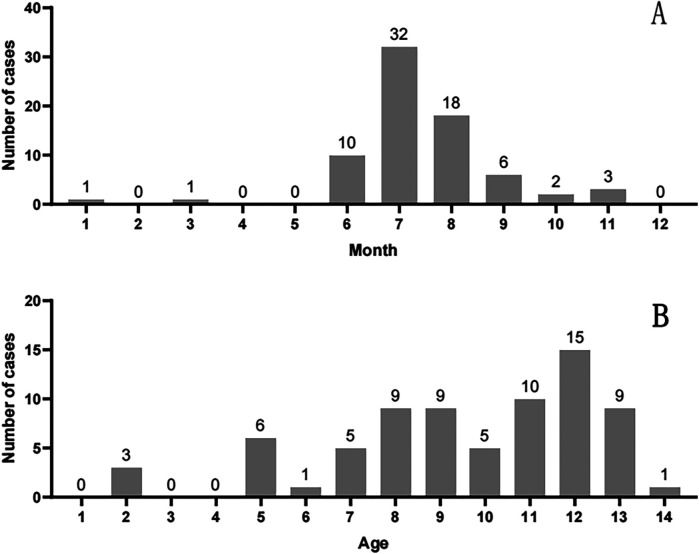
Seasonal **(A)** and age **(B)** distribution of pediatric mushroom poisoning.

Cases peaked during the core rainy season (June–October), when provincial rainfall exceeds 150 mm/month and humidity averages >80%. This period drives explosive mushroom growth across Yunnan, directly explaining the observed summer (June–August) and autumn (September–October) clustering (Figure 2A). Children aged ≥7 years accounted for 78.1% of cases (57/73), reflecting increased foraging autonomy and reduced supervision in school-aged children (Figure 2B).

### Mushroom species identification

**Figure 3:**
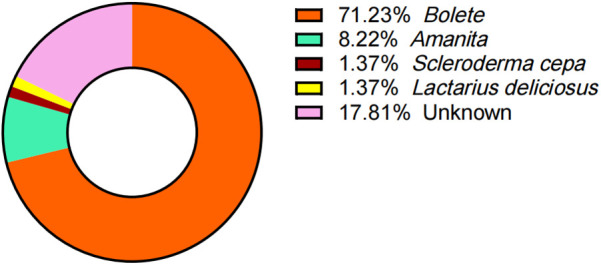
Causative Species or presumed toxin sources.

Despite comprehensive efforts to document the mushroom species ingested, 13 pediatric cases remained unidentified due to unclear recollection by children and guardians. Among the 60 cases with confirmed mushroom taxonomy, the implicated species were distributed as follows: *Boletus* species (*n* = 52, 71.23%), *Amanita spp.* (*n* = 6, 8.22%), and 1 case each of *Scleroderma cepa* and *Lactarius deliciosus* (Figure 3).

### Clinical manifestations

We concluded the symptom profile of 73 children with mushroom poisoning upon hospital admission in Table 1. Gastrointestinal toxicity predominated (72.6%), with nausea and vomiting being the most frequent symptoms. Neurotoxic effects were notable (69.9%), particularly visual/auditory hallucinations (50.7%) and vertigo (39.7%). The low incidence of fever (16.4%) suggests a toxin-specific pattern distinct from systemic infections.

**Table 1:** Clinical symptoms in 73 pediatric patients with mushroom poisoning (symptoms may derive from multiple toxins due to mixed mushroom ingestion.

Symptoms	*n* (%)
Fever	12 (16.44%)
Gastrointestinal	53 (72.60%)
Nausea	53 (72.60%)
Vomiting	47 (64.38%)
Diarrhoea	12 (16.44%)
Abdominalpain	25 (34.25%)
Neurological/Psychiatric	51 (69.86%)
Hallucinations	37 (50.68%)
Vertigo	29 (39.73%)
Spasms	6 (8.22%)

Dominant species listed represents primary but not exclusive exposure).

### Hepatic injury and prognostic factors

**Figure 4:**
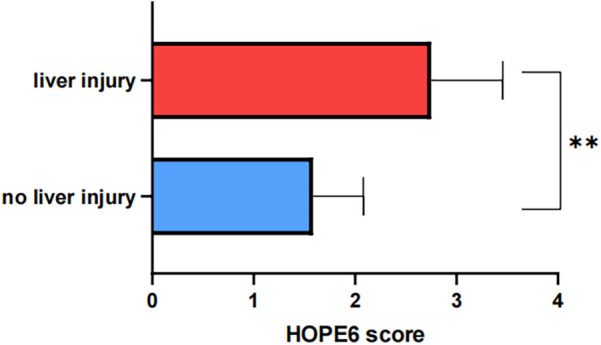
Difference in HOPE6 score between liver injury and no liver injury group (“*” means *p* < 0.05; “**” means *p* < 0.01).

We recognized that hepatic impairment is one of the most prominent features of mushroom poisoning. Therefore, patients were stratified into two groups based on the presence or absence of liver injury, and comprehensive statistical analyses were performed on clinical parameters between these groups. As demonstrated in Table 2, the liver injury group exhibited significantly higher HOPE6 scores and markedly elevated liver function/coagulation-related laboratory indices (ALT, AST, LDH, TBIL, APTT, PT) compared to the no liver injury group (all *p* < 0.05), and showed the difference in Figure 4. Notably, all fatal cases were exclusively observed in the liver injury group. Additionally, the liver injury group presented more severe gastrointestinal manifestations [diarrhoea (87.5% vs. 7.69%, *P* < 0.01), abdominalpain (100% vs. 26.15%, *p* < 0.01), FOB (75% vs. 13.85%, *p* < 0.01)], whereas the no liver injury group showed a higher incidence of hallucinations (56.92% vs. 0%, *P* = 0.02). No statistically significant differences were found between the two groups in age, sex, BMI, or hospitalization length (*p* > 0.05).

**Table 2:** Comparison of demographic characteristics, clinical manifestations, laboratory parameters and outcomes between hepatic and no hepatic impairment groups in pediatric patients.

Variables	No liver injury (*n* = 65)	Liver injury (*n* = 8)	*P*
Age	9.75 ± 2.81	8.38 ± 3.5	0.273
Male	31 (47.69%)	4 (50%)	0.597
BMI	18.01 ± 3.44	15.82 ± 1.18	0.056
Hospitalization length	3.23 ± 2.29	3.88 ± 3.27	0.836
HOPE6 score			<0.01
1	27 (41.54%)	0	
2	38 (58.46%)	3 (37.5%)	
3	0	4 (50%)	
4	0	1 (12.5%)	
ALT	17.54 ± 9.75	4,029.16 ± 3,796.28	<0.01
AST	25.62 ± 9.33	4,127.7 ± 4,185.49	<0.01
LDH	241.88 ± 55.76	3,140.25 ± 3,096.48	<0.01
TBIL	10.4 ± 5.21	88.83 ± 104.14	<0.01
DBIL	3.7 ± 2.1	36.33 ± 33.42	<0.01
UBIL	6.74 ± 3.68	54.2 ± 72.51	<0.01
ALB	43.12 ± 3.75	31.95 ± 5.54	<0.01
CK-MB	17.28 ± 12.74	37.87 ± 26.4	0.09
Cr	44.44 ± 10.54	64.5 ± 43.51	0.305
BUN	5.23 ± 3.89	4.18 ± 1.8	0.203
UA	330.46 ± 85.75	353.75 ± 154.74	0.825
Na^+^	139.14 ± 2.49	134.75 ± 5.42	0.006
K^+^	4.02 ± 0.31	4.07 ± 0.26	0.619
APTT	39.71 ± 5.46	106.91 ± 57.09	<0.01
PT	13.96 ± 0.96	65.24 ± 49.28	<0.01
Fever	8 (12.31%)	4 (50%)	0.021
Nausea	45 (69.23%)	8 (100%)	0.066
Vomiting	39 (60%)	8 (100%)	0.023
Diarrhoea	5 (7.69%)	7 (87.5%)	<0.01
Abdominalpain	17 (26.15%)	8 (100%)	<0.01
Hallucinations	37 (56.92%)	0	0.02
Vertigo	25 (38.46%)	4 (50%)	0.396
Spasms	4 (6.15%)	2 (25%)	0.127
Coagulation disorders	0	5 (62.5%)	<0.01
FOB	9 (13.85%)	6 (75%)	<0.01
Hepatomegaly	2 (3.08%)	5 (62.5%)	<0.01
Mortality	0	4 (50%)	<0.01

### Mortality analysis

**Figure 5:**
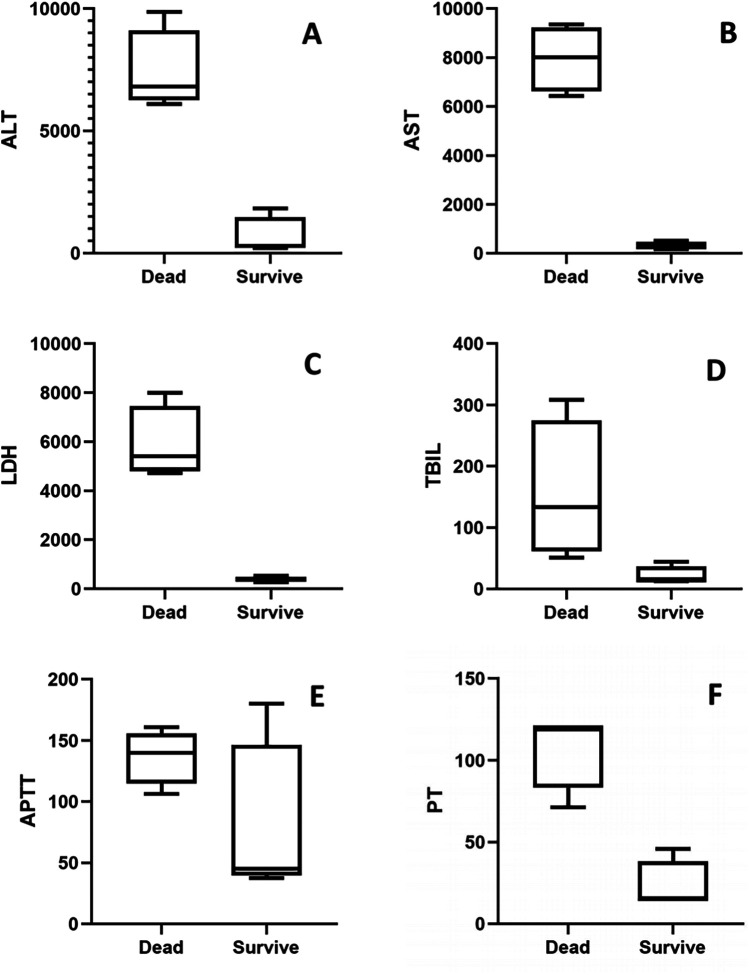
Comparison of liver and coagulation markers between dead and survive chilren: **(A)** ALT, **(B)** AST, **(C)** LDH, **(D)** TBIL, **(E)** APTT, **(F)** PT.

Furthermore, we conducted a subgroup analysis comparing liver function and coagulation profiles between dead and survive cases within the liver injury cohort. As shown in Figure 5, dead cases exhibited 25- to 40-fold elevations in liver function parameters (ALT, AST, LDH, TBIL in Figure 5A–D) and 2- to 5-fold increases in coagulation indices (APTT, PT in Figures 5E,F) compared to survivors.

We have summarized the key demographic and clinical characteristics of the four dead cases in Table 3. The patients were aged 2, 12, 5, and 10 years, with a male-to-female ratio of 1:3 (1 male and 3 females). The hospitalization duration ranged from 1 to 3 days, and BMI values fell between 15.5 and 17.8 kg/m². The implicated mushroom species included *Boletus* (*n* = 1), *Amanita* (*n* = 1), and unidentified species (*n* = 2). All exposures clustered in June and July, with HOPE6 scores of 3 or 4 observed across these cases.

**Table 3:** Demographic characteristics of deceased pediatric patients.

Patient ID	Age	Sex	Hospitalization length	BMI	Mushroom species	Month	HOPE6 score
1	2	Male	2 days	15.5	*Bolete*	7	3
2	12	Female	3 days	15.6	*Amanita*	6	4
3	5	Female	1 day	17.0	unknown	7	3
4	10	Female	1 day	17.8	unknown	7	3

Supplement: The HOPE6 scoring criteria.

## Discussion

The summer-autumn predominance (June–July) aligns with monsoon-driven mushroom proliferation, mirroring patterns in subtropical Asia but contrasting with temperate regions where spring peaks prevail (5, 11, 12). Key epidemiological patterns align with global pediatric cohorts (1, 2): Seasonal clustering during monsoon months (June–October in Yunnan vs. May–October in Thailand); Predominance of school-aged children (≥7 years: 78.1% here vs. 74% in Thailand); High neurotoxicity incidence (69.9% vs. 61% in Thailand). Additionally, the high incidence in older children reflects reduced parental supervision and increased foraging autonomy. Consistent with previous studies (13), the majority of pedspiatric mushroom poisoning cases involved *Boletus* species, that may reflect their widespread culinary popularity globally despite inherent toxicity risks.

Severe gastrointestinal symptoms (diarrhea, abdominal pain, positive fecal occult blood) in the liver injury group align with the pathological mechanisms of amatoxin-containing mushrooms (e.g., *Amanita*), which damage intestinal epithelial cells and trigger systemic inflammatory responses (14–16). Intriguingly, hallucinations occurred in 56.9% of non-liver injury cases, possibly linked to neurotoxic species. Clinicians must remain vigilant for atypical neurological symptoms that may mask underlying hepatotoxicity.

Our findings highlight the critical need to contextualize symptoms within toxin-specific syndromes, rather than attributing them universally to mushroom toxicity. Although Boletus species dominated our cohort (71.23%), gastrointestinal manifestations (e.g., vomiting, diarrhea) may stem from heterogeneous etiologies. Firstly, true toxin-mediated injury: Enterotoxins (e.g., amatoxins in Amanita; gastrointestinal irritants in some Boletus) directly damaging intestinal epithelium (14, 16). Secondly, non-toxin mechanisms: Bacterial contamination, inadequate cooking of inherently edible species (17). This is exemplified by our single *Lactarius deliciosus* case: the child developed vomiting after eating hastily stir-fried mushrooms—a presentation consistent with thermolabile toxin exposure rather than inherent toxicity.

In regions like Yunnan with high mycological diversity and mixed foraging practices, symptoms represent composite toxidromes. Forcing cases into discrete syndromes risks oversimplifying mixed exposures. We acknowledge the utility of the toxin-syndrome model for pure exposures, but co-ingestion of minor species may modify presentations—a limitation absent in controlled single-species studies (17).

Consistent with prior studies, the HOPE6 score effectively predicts adverse outcomes in mushroom-poisoned patients (10, 13). Notably, while a score ≥2 is prognostic of poor outcomes in adults (10), pediatric patients exhibit a distinct threshold, with scores ≥3 indicating significantly elevated risks. We also observed a considerable proportion of pediatric patients in the non-liver injury group with delayed symptom onset (>6 hours post-ingestion). In our study, 41.1% (30/73) of non-hepatic injury cases exhibited E6 positivity without progression to severe outcomes (0% mortality). Unlike in adults where delayed onset strongly correlates with adverse outcomes (15), this temporal pattern did not predict poor prognosis in children. This discrepancy in clinical significance may partially explain the divergent HOPE6 score thresholds between adults (≥2) and pediatric populations (≥3), potentially reflecting age-dependent variations in toxin metabolism or compensatory physiological responses. We hypothesize that developmental differences in gastrointestinal motility and enterotoxin metabolism may attenuate the predictive value of E6 in children. Pediatric patients exhibit accelerated gastric emptying and intestinal transit compared to adults (18–20), potentially altering toxin absorption kinetics for certain mushrooms. This may decouple symptom latency from toxin load severity in children.

This retrospective study has several limitations. Critical variables (including the quantity of mushroom ingested, cooking methods, toxin detection, and occurrence of clustered poisoning events) were not analyzed due to incomplete data records. Our inability to quantify ingested mushroom doses stems from inherent cultural and developmental constraints: Collective dining practices prevent individual portion tracking; And pediatric recall is unreliable during acute neurotoxicity. Moreover, homogeneous cooking methods—exclusively stir-frying or boiling with most of cases involving both techniques—precluded meaningful analysis of culinary mitigation effects on toxins. Additionally, although our cohort represents one of the largest pediatric-specific analyses of mushroom poisoning in the region, the sample size (*n* = 73) limits the statistical power for subgroup analyses, particularly for mortality risk modeling (4 deaths). This may affect the generalizability of our findings to broader populations. Future multicenter collaborations are essential to validate the HOPE6 pediatric threshold (≥3) and establish robust prognostic markers.

Future studies should involve multicenter prospective cohorts to conduct multivariate regression analysis of the age-dependent HOPE6 threshold and mortality predictors identified here. These works effort will standardize toxin identification, document ingested quantities, and assess interventions—ultimately enabling multivariate regression models for pediatric-specific risk stratification.

## Conclusion

This 15-year retrospective cohort study delineates the epidemiological and clinical characteristics of pediatric mushroom poisoning in part of Southwest China. Pediatric mushroom poisoning predominantly occurs during the summer and autumn seasons, coinciding with peak mushroom growth, and primarily affects school-aged children. *Boletus* species account for the majority of poisoning cases, followed by *Amanita*, while intoxications from other mushroom varieties are relatively rare. Severe hepatic impairment and coagulation dysfunction are critical determinants of mortality in pediatric mushroom poisoning. Furthermore, the HOPE6 score ≥3 predicts adverse clinical outcomes.

## Data Availability

The raw data supporting the conclusions of this article will be made available by the authors, without undue reservation.
