# Supplementary material for: Epidemiology and clinical aspect of pediatric mushroom poisonings: a 15-year retrospective analysis
Source: Front Pediatr. 2025 Sep 18;13:1621891. doi: 10.3389/fped.2025.1621891 (PMC12488608; doi:10.3389/fped.2025.1621891)
Supplement: Supplementary file 1 [file Datasheet1.pdf]

The HOPE6 Scoring Criteria

**1.History (H)**	Confirms mushroom consumption history, time from ingestion to symptom onset, and whether others who consumed the same mushrooms have fallen ill. It also checks for retained mushroom samples or photos, and alcohol consumption alongside the mushrooms.
**2.Organ Damage (O)**	Assesses signs of instability in vital signs and organ dysfunction, particularly in the liver, kidneys, and coagulation system.
**3.Picture Identification (P)**	Identifies the type of mushroom consumed through pictures or physical samples, often with the help of mycology experts.
**4.Eruption of Symptom >6h (E6)**	Evaluates whether symptom onset occurred more than six hours after mushroom ingestion.
